# Editorial: Plant-virus interactions: crop resistance in focus

**DOI:** 10.3389/fpls.2023.1354316

**Published:** 2024-01-08

**Authors:** Jiban Kumar Kundu, Heng-Mu Zhang, Supriya Chakraborty

**Affiliations:** ^1^ Plant Virus and Vector Interactions-Centre for Plant Virus Research, Crop Research Institute, Prague, Czechia; ^2^ Laboratory of Virology-Centre for Plant Virus Research, Institute of Experimental Botany of the Czech Academy of Sciences, Prague, Czechia; ^3^ Institute of Virology and Biotechnology, Zhejiang Academy of Agricultural Sciences, Hangzhou, China; ^4^ Molecular Virology Laboratory, School of Life Sciences, Jawaharlal Nehru University, New Delhi, India

**Keywords:** resistance, crop, virus, host-virus interaction, defence mechanisms

Viral pathogens cause physiological changes in infected plants that lead to different types of compatible host-virus interactions, resulting in infection of the host and the development of diseases. Crop damages caused by viral diseases cause substantial annual losses exceeding $30 billion (Jones, 2021).

A compatible interaction leads either to either host susceptibility or resistance. In susceptible hosts the virus infects and replicates, causing symptoms of various severity and types. Conversely, host resistance involves limited or no viral replication, resulting in localized or no apparent disease symptoms (Cooper and Jones, 1983; Kang et al., 2005). In general, plant resistance to viral diseases is typically inherited genetically and can be improved through breeding or genetic modification. This involves blocking or hindering the steps of the virus life cycle, including virus replication within cells, cell-to-cell movement, and long-distance systemic movement within a non-permissive host plant. As intercellular pathogens, viruses utilize various host proteins to regulate pathogenesis for compatible interaction with the host. In response, plants have evolved various defence systems to enhance the incompatible interaction, leading to host resistance. Plant antiviral defence mechanisms include host R gene-mediated responses (Sett et al., 2022), RNA silencing or RNA interference (Baulcombe, 2004) and manipulation of host susceptibility factors (Truniger and Aranda, 2009). Some of these mechanisms have been targeted to acquire plants (crops) resistance, with varying degrees of success in different crops and viruses. This Research Topic, ‘Plant-Virus Interactions: Crop Resistance in Focus”, brings together both original research articles and reviews that address some of the key challenges in developing plant resistance to viruses.

An integrative approach to disease resistance would involve investigating the role of different phytohormones in plant-virus interactions and disease development. Gnanasekaran et al. show how in potyvirus (potato virus Y-PVY) the viral protein modulates auxin homeostasis to promote disease development. The non-structural viral NIa-pro gene promotes symptom development by interacting with a host indole-3-acetic acid amido synthetase (IAAS). This research has also shown that symptoms and virus accumulation are reduced in IAAS-silenced plants, suggesting that the auxin-mediated defence response occurs via depletion of the free auxin pool through interaction with IAAS.

Recessive resistance, often based on mutation or knock-out of the host susceptibility factor, has been shown to be an effective means of developing resistance to various plants and viruses. The best known of these factors is the translation initiation factor eIF4E, which was originally described for resistance to potyviruses in various crops. It was later shown that resistance mediated by eIF4E is also effective against other viruses such as bymoviruses, potexviruses, tritimoviruses, ipomoviruses, carmoviruses, carlaviruses and cucumoviruses. In their review article, Zlobin and Taranov investigated the role of different isoforms of eIF4E in the interaction between plant viruses and resistance of potyviruses based on the mechanism of loss of susceptibility. It is suggested that understanding the interaction between the eIF4E isoforms in plant-potyvirus interactions could also be used for the development of resistance to closely related viruses.

Genomic variation of viruses caused by mutation through recombination and the acquisition of additional genomic components is common in plant viruses, where the consensus sequence changes in response to selection pressure. Liebe et al. reveal details of the mechanism of resistance breakdown against Beet necrotic yellow vein virus (BNYVV) and suggest that genomic flexibility, specifically mutations in the virus genome together with the encoding of the pathogenicity factor P26 in RNA5, enables the overcoming of resistance mediated by an R gene, Rz1, in sugar beet. This research has also shown that genotypes with two R genes (Rz1+Rz2) can confer resistance to BVYVV and emphasizes the need for a comprehensive interdisciplinary approach to identify new resistance candidate genes.

Wu et al. investigated the modification of epigenetic mark histone H3K9me3 of the rice genome during infection with rice grassy stunt virus (RGSV), which provides target genes for resistance to the virus. Further, they showed that after RGSV infection, H3K9me3 modification significantly changed at the whole genome level and affected the expression of some genes related to the hormone pathway, such as cytoplasmic tyrosine kinase (CTK: cZOGT1, CKX11) and brassinosteroid (BR: DET2;2, CYP734A4), and these might be the putative target genes for resistance. Therefore, genome-wide gene function analysis seems to be a valuable tool to identify important resistance genes against viral infections.

The contributions presented in this Research Topic are valuable for the development of resistance of plants and crops against viruses. The results will contribute to a better understanding of plant resistance mechanisms and to the improvement of plants in combating viral diseases.

## Author contributions

JK: Writing – original draft, Writing – review & editing. HZ: Writing – review & editing. SC: Writing – review & editing.
